# Periodontal disease and chronic kidney disease among Aboriginal adults; an RCT

**DOI:** 10.1186/s12882-015-0169-3

**Published:** 2015-10-31

**Authors:** Lisa Jamieson, Michael Skilton, Louise Maple-Brown, Kostas Kapellas, Lisa Askie, Jaqui Hughes, Peter Arrow, Sajiv Cherian, David Fernandes, Basant Pawar, Alex Brown, John Boffa, Wendy Hoy, David Harris, Nicole Mueller, Alan Cass

**Affiliations:** Indigenous Oral Health Unit, University of Adelaide, Adelaide, Australia; Boden Institute, Unversity of Sydney, Sydney, Australia; Menzies School of Health Research, Charles Darwin University, Darwin, Australia; Clinical Trials Centre, Unversity of Sydney, Sydney, Australia; Alice Springs Renal Unit, Northern Territory Government, Alice Springs, Australia; South Australian Health and Medical Research Institute, Adelaide, Australia; Central Australian Aboriginal Congress, Alice Springs, Australia; Centre for Chronic Disease, University of Queensland, Brisbane, Australia; Sydney Medical School, University of Sydney, Sydney, Australia

## Abstract

**Background:**

This study will assess measures of vascular health and inflammation in Aboriginal Australian adults with chronic kidney disease (CKD), and determine if intensive periodontal intervention improves cardiovascular health, progression of renal disease and periodontal health over a 24-month follow-up.

**Methods:**

The study will be a randomised controlled trial. All participants will receive the periodontal intervention benefits, with the delayed intervention group receiving periodontal treatment 24 months following baseline. Inclusion criteria include being an Aboriginal Australian, having CKD (a. on dialysis; b. eGFR levels of <60 mls/min/1.73 m^2^ (CKD Stages 3 to 5); c. ACR ≥30 mg/mmol irrespective of eGFR (CKD Stages 1 and 2); d. diabetes plus albuminuria (ACR ≥ 3 mg/mmol) irrespective of eGFR), having moderate or severe periodontal disease, having at least 12 teeth, and living in Central Australia for the 2-year study duration. The intervention involves intensive removal of dental plaque biofilms by scaling, root-planing and removal of teeth that cannot be saved. The intervention will occur in three visits; baseline, 3-month and 6-month follow-up. The primary outcome will be changes in carotid intima-media thickness (cIMT). Secondary outcomes will include progression of CKD or death as a consequence of CKD/cardiovascular disease. Progression of CKD will be defined by time to the development of the first of: (1) new development of macroalbuminuria; (2) 30 % loss of baseline eGFR; (3) progression to end stage kidney disease defined by eGFR <15 mLs/min/1.73 m^2^; (4) progression to end stage kidney disease defined by commencement of renal replacement therapy. A sample size of 472 is necessary to detect a difference in cIMT of 0.026 mm (SD 0.09) at the significance criterion of 0.05 and a power of 0.80. Allowing for 20 % attrition, 592 participants are necessary at baseline, rounded to 600 for convenience.

**Discussion:**

This will be the first RCT evaluating the effect of periodontal therapy on progression of CKD and cardiovascular disease among Aboriginal patients with CKD. Demonstration of a significant attenuation of CKD progression and cardiovascular disease has the potential to inform clinicians of an important, new and widely available strategy for reducing CKD progression and cardiovascular disease for Australia’s most disadvantaged population.

**Trial registration:**

This trial is registered with the Australian New Zealand Clinical Trial Registry ANZCTR12614001183673.

## Background

### Aboriginal Australian health

Aboriginal Australians experience poor health relative to their non-Aboriginal counterparts [1]. They have 15–20 years shorter life expectancy, much higher levels of chronic diseases, and are more likely to experience disability and reduced quality of life due to ill health. There is a heavy burden of infectious diseases as well as high rates of metabolic risks among the Aboriginal population [2]. Along with other chronic conditions, dental disease and chronic kidney disease levels in the Aboriginal population are equivalent to those in the poorest nations [3, 4]. There are many reasons why such conditions are common. Financial barriers are frequently cited, together with lack of service access, cultural factors, global risk factors and social determinants. While recognising their importance, this proposal does not address these influences, but rather focuses on the role of oral health, specifically periodontal disease, in Aboriginal renal health.

### Chronic kidney disease (CKD); the problem

CKD is characterised by a progressive loss of renal function over a period of months or years culminating with end stage kidney disease (ESKD), when a person will require ongoing dialysis or a kidney transplant to stay alive, being the final stage of this progressive loss [5]. Like most other chronic diseases, the burden of CKD and consequent ESKD in Australia is increasing. It is estimated that approximately 12 % of Australians have either reduced kidney function or other markers of kidney damage [6]. There is clear evidence across the spectrum of CKD of an excess burden of premature comorbidity and mortality due to cardiovascular disease (CVD). CKD contributes to approximately 15 % of total Australian hospitalisations, with current medical costs for ESKD patients amounting to more than $1 billion annually [7]. Overall, patients with CKD have a mortality rate around 4 times that of their non-CKD counterparts [8].

### CKD; the measurement

Common measures used in the assessment of CKD are the urinary albumin-to-creatinine ratio (ACR) and glomerular filtration rate (GFR). Albuminuria (or microalbuminuria) is defined as urine ACR ≥3 and ≤30 mg/mmol, while proteinuria (macroalbuminuria) is defined as ACR ≥ 30 mg/mmol [9]. Because GFR cannot be directly measured in usual clinical practice, it is estimated from equations using serum creatinine, age, sex and body size [10]. Estimated GFR is referred to as eGFR. Both reduced eGFR and level of proteinuria are independent risk factors for CVD morbidity and mortality, and for progression of ESKD.

### CKD among Aboriginal Australians

Findings from the 2012–13 Australian Aboriginal and Torres Strait Islander Health Survey indicate that the prevalence of CKD amongst Australian Indigenous adults is approximately 18 % [11]. The National Biomedical Risk Factor Survey (which included approximately 3300 Indigenous adults) revealed that the prevalence of CKD was 34 % in remote locations, and that approximately 53 % of people with diabetes had signs of CKD. In Central Australia, more than 40 % of Aboriginal Health Service attendees were reported as having micro or macroalbuminuria and 40 % were reported as having reduced eGFR [12]. Dialysis is the leading cause of hospitalisation for Aboriginal Australians—constituting 42 % of all admissions [8]. Recent literature suggests that the prevalence nationwide of Aboriginal Australians with ESKD resulting in renal replacement therapy is around 10 times that of non-Aboriginal Australians [4]. Aboriginal Australians with ESKD are younger than their non-Aboriginal counterparts and there is high comorbidity. There is regional variability, with Aboriginal Australians living in remote Northern Territory communities having 20 times the prevalence of renal replacement therapy than remote-living non-Aboriginal Australians [13, 14]. Renal replacement therapy is strongly associated with socio-economic disadvantage [15, 16].

### Periodontal disease

Periodontal disease is inflammation of the tissues surrounding teeth and results from a complex interplay between bacteria and host risk factors. Periodontal organisms flourish in the presence of inflammation, enabling them to invade host tissues and to gain direct access to the circulation [17]. The constant exposure of the vasculature to these pathogens provides an opportunity for endothelial inflammatory activation and functional impairment [18]. Clinically, this manifests as deepening of the epithelial attachment around teeth, loss of periodontal attachment and, ultimately, tooth loosening. The total burden of this infection may be significant; specifically, it may account for a portion of the proposed risk for CKD and other systemic conditions that share an underlying inflammatory response as a common component of pathogenesis [17].

### Periodontal disease and Aboriginal Australians

Although representative national-level data are unavailable, estimates from convenience samples indicate that periodontal disease is a substantial problem among Aboriginal Australians. The prevalence of periodontal disease among Aboriginal participants seeking care at the Redfern Aboriginal Medical Service was around 60 % [19], while the prevalence was approximately 90 % in a recent convenience sample in the Northern Territory [20].

### CKD and periodontal disease

Periodontal disease has emerged as a non-traditional risk factor for CKD [21]. In nationally-representative cross-sectional surveys, there is increasing evidence of associations between CKD and periodontal disease, with a higher proportion of those with CKD having periodontal disease and vice versa [22–24]. Biological plausibility for considering periodontal disease as a CKD risk factor is derived from the potential role of the inflammatory response to periodontal disease in the chronic systemic inflammatory burden (for example, increased C-reactive protein (CRP) levels) associated with CKD [25–28]. The local tissue destructive immuno-inflammatory response to periodontal pathogens, their products and inflammatory cytokines are believed to contribute to the chronic systemic inflammatory burden of periodontal disease [29, 30].

### Can CKD progression be reduced by periodontal therapy?

Few studies have investigated the effect of periodontal treatment on kidney function. In a prospective cohort of haemodialysis patients in Thailand, successful non-surgical periodontal treatment resulted in marked reductions in CRP (mean change −3.2 mg/L) after two months [31]. A similar reduction in CRP following periodontal treatment was reported among patients with CKD in an Italian-based study (mean change −2.1 mg/L) [31]. Graziani and colleagues, in a prospective cohort including 20 healthy people, reported that successful periodontal treatment improved cystatin C levels but had no effect on eGFR after 3 months (mean change −1.4 mL/min/1.73 m^2^) [32]. In a non-randomised clinical trial involving 40 people, Artese et al. reported that periodontal therapy significantly improved eGFR among patients with CKD (mean change 4.2 mL/min/1.73 m^2^), whereas there were no differences in serum creatinine [33]. A reduction in proteinuria was obtained following a periodontal intervention among 11 patients with IgA nephropathy, but all patients were children [34]. There are substantial shortcomings with each of these studies; all have small sample sizes, most have only one periodontal therapy session and none were randomised controlled trials. There are also considerable differences in patient populations; the severity of CKD among our Aboriginal group will be greater, suggesting the possibility of increased efficacy of the periodontal treatment. Shortcomings aside, the evidence suggests that periodontal health may improve renal health, warranting a more robust clinical trial study design.

### Can CVD progression be reduced by periodontal therapy?

There is some evidence that CVD is influenced by periodontal health. Piconi et al. reported a diminution in carotid intima media thickness (cIMT; an established, non-invasive measure of atherosclerotic burden previously validated against histology specimens. cIMT is associated with cardiac risk factors, and predicts incident cardiovascular events) [35–39] 12 months following periodontal treatment among 35 otherwise healthy adults (mean change −0.12 mm, *P* < 0.001) [38]. In our recent periodontal intervention among Aboriginal adults in the Northern Territory, cIMT decreased in those randomised to the periodontal intervention (−0.026 mm [95 % CI −0.048, −0.003], *P* = 0.03) compared to control group [39]. The magnitude of the reduction in carotid IMT with periodontal intervention in this trial was equivalent to the effects of reversal of 4 years of aging, 8 kg/m^2^ lower body mass index or 25 mmHg lower systolic blood pressure. Mercanoglu and colleagues provided the first evidence that treating periodontal disease results in a functional improvement in cardiovascular status [40]. Among 54 otherwise healthy patients, flow mediated dilation (FMD)—a measure of endothelial function representing an important early stage of atherosclerosis [41], with its presence in coronary or peripheral vessels constituting an independent predictor of cardiovascular events [42], improved from 8.4 % to 17.7 % 6 weeks post periodontal treatment (*P* < 0.01). In an RCT, Tonetti et al. demonstrated improved FMD after 2 months (absolute difference 0.9 %; *P* = 0.02) and 6 months (absolute difference 2.0 %; *P* < 0.001) for the intervention group who received intense periodontal therapy [43]. The degree of improvement was associated with improvement in measures of periodontal disease (r = 0.29, *P* = 0.003).

### Aims and hypotheses

The goals are to assess levels of periodontal disease among a group of Aboriginal adults with CKD and to determine if comprehensive periodontal therapy might reduce progression of CKD, non-invasive measures of cardiovascular disease (CVD) or CKD/CVD-related death amongst people with CKD over 6 to 24 months. The specific aims and hypotheses are:

- Aim 1: To describe the extent and severity of periodontal disease in a group of Aboriginal adults with CKD.
- *Hypothesis: The extent and severity of periodontal disease in an Aboriginal population with CKD will be high compared with national-level indicators.*
- Aim 2: To examine the effects of a comprehensive periodontal intervention among an Aboriginal population with CKD.
- *Hypothesis: Exposure to a comprehensive periodontal intervention will reduce progression of CKD, non-invasive measures of CVD or CKD/CVD-related death amongst those with CKD.*

## Methods

### Study design

**Fig. 1:**
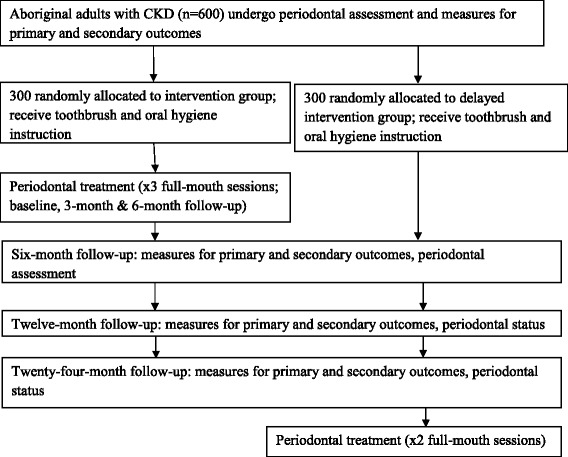
Study plan schema

This will be a delayed intervention RCT, with all participants ultimately receiving the periodontal intervention benefits (Fig. 1).

### Ethical approval

The study was approved by the University of Adelaide Human Research Ethics Committee and the Central Australian Human Research Ethics Committee. Written informed consent will be obtained from study participants before their involvement in the trial.

### Inclusion criteria

Aboriginal people aged 18+ years who have lived in the study location for 2+ years, and who plan to live at their current location for the next 2 years, with CKD (i. on dialysis; ii. eGFR levels of <60 mls/min/1.73 m^2^ (CKD Stages 3 to 5); iii. ACR ≥30 mg/mmol irrespective of eGFR (CKD Stages 1 and 2); iv. diabetes plus albuminuria (ACR ≥ 3 mg/mmol) irrespective of eGFR), with at least 12 teeth at baseline and having moderate or severe periodontal disease will be invited to take part in the study. Periodontal disease will be assessed using the US Centers for Disease Control and Prevention and the American Academy of Periodontology definitions, whereby a case of moderate periodontitis is considered as the presence of either two sites between adjacent teeth with 4 mm + attachment loss or at least two such sites with 5 mm + pockets, and severe periodontitis as having at least two sites between adjacent teeth with 6 mm + attachment loss and at least one 5 mm + pocket [44].

### Randomisation

Randomisation will be developed by biostatisticians at the Australian Research Centre for Population Oral Health using a random number generator. Randomly selected block sizes of 4, 6 and 8 will be used. This will guarantee an equal number of participants in each intervention arm within the blocks, with any differences in confounders and other characteristics being then due to chance. Randomisation will be done using field staff laptop computers. The field staff will be required to register the participants in the trial prior to identifying the randomised group. These data will be unable to be altered, thus allowing the randomisation process to be audited.

### Periodontal intervention

The periodontal intervention will be based on the technique described by Tonetti and colleagues [43]. This involves intensive removal of subgingival dental plaque biofilms by scaling, root-planing and removal of teeth that cannot be saved, following administration of local anaesthesia. The intervention will be performed by registered oral health professionals and will occur in three visits; baseline, 3 months and 6 months post-baseline.

### Location

The study will be undertaken in Alice Springs, Northern Territory of Australia.

### Engagement, recruitment and retention

This study will rely on commitment from the Alice Springs primary and tertiary health care sector. Conveniently, the Alice Springs Renal Dialysis Unit is located a few metres from the Alice Springs-based Northern Territory Oral Health Services. Specific recruitment strategies will include: establishing service agreements with the key Aboriginal community-controlled health and mainstream government health services in Alice Springs; liaising with community champions previously involved in Alice Springs-based research projects; engaging with key community stakeholder groups; encouraging word-of-mouth spread of knowledge about the study; advertisements in local newspapers and radio shows; flyers in post boxes in high-density Aboriginal locations and presentations made to community groups. A passive snowballing technique will also be employed, with participants asked to contact any Aboriginal friends, family and peers who may be interested in partaking. Retention strategies will involve: (1) employing staff who are committed to following up participants despite challenges in doing so; (2) ensuring participants are contacted on a regular basis to check accuracy of contact details; (3) maintaining relationships with appropriate stake holders; (4) ascertaining contact details of three key personnel who may know the whereabouts of participants should the study team be unable to contact them; (5) sending birthday and Christmas cards to participants; and (6) faciliating one-on-one relationships between study staff and participants, with study staff ideally seeing each of their participants for each phase of the research. We will allow 2 years for recruitment.

### Follow up

Participants will be followed for 24 months after randomisation. Because this is a pragmatic study evaluating the delivery of periodontal care, rather than therapeutic agents, an open design is necessary. Appropriate care will be taken to ensure standardised, and where feasible, blinded outcome evaluation—“PROBE” design [45]. During follow-up, all study participants will attend clinic visits at 6 months, 12 months and at 24 months.

### Sample size

Based on a recent study of Australian Aboriginal adults with periodontal disease, it was estimated that a sample size of 472 would be necessary to detect a difference in cIMT of 0.026 mm (SD 0.09) [46] at the significance criterion of 0.05 and a power of 0.80. Allowing for 20 % attrition, 592 participants would be necessary at baseline, rounded to 600 for convenience.

### Measures

*Primary outcome:* The primary outcome will be change in cIMT over 24 months from randomization.

*Secondary outcomes;* will include progression of CKD, death as a consequence of CKD/CVD, periodontal status, endothelial function, arterial stiffness, measures of inflammation and infection, CVD risk factors (blood pressure, total cholesterol, HDL-cholesterol, HbA1c), CV events (myocardial infarction, coronary revascularisation procedures, ischaemic cerebrovascular disease, peripheral vascular disease, ischaemic stroke) and oral health-related quality of life. Progression of CKD will include time to the development of the first of: (1) new development of macroalbuminuria (defined as ACR ≥30 mg/mmol); (2) 30 % loss of baseline eGFR (defined as a reduction of eGFR of 30 % or more from baseline and eGFR of less than 60mLs/min/1.73 m2); (3) progression to ESKD defined by eGFR <15 mLs/min; (4) progression to ESKD defined by commencement of renal replacement therapy.

### Data collection techniques

Both clinical and self-report information will be gathered in this study.

*Clinical*: Each participant will receive CKD/CVD, cardio-metabolic-related and dental assessments at four time points throughout the study (baseline, 6 months, 12 months and 24 months post randomization).

#### CKD/CVD and cardio-metabolic-related assessments

Markers of renal function will be assessed by ACR and eGFR. eGFR will be assessed using the CKD-EPI formula: eGFR = 141 × min(Scr × 0.0113/k, 1)^α^ × max(Scr × 0.0113/k, 1)^−1.209^ × 0.993^Age^ × 1.018 [if female], where Scr is serum creatinine concentration in μmol/L, k is 0.7 for females and 0.9 for males, α is −0.329 for females and −0.411 for males, min indicates the minimum of Scr/k or 1 and max indicates the maximum of Scr/k or 1. Maple-Brown and colleagues demonstrated that the correction factor “if black” is not applicable to Aboriginal Australians [47].

Markers of systemic inflammation will be assessed by CRP, serum amyloid A, fibrinogen, neutrophils and total IgG. Standard Framingham risk factors will be assessed (non-fasting lipids, blood pressure, diabetes control (HbA1c), BMI and tobacco smoking) as well as waist-hip ratio and medications including ACE inhibitors and angiotensin receptor blockers. Peripheral blood pressure will be measured and central pressure calculated (SphygmoCor XCEL).

Markers of cardiovascular health will be measured by: (1) cIMT; (2) endothelial function; (3) arterial stiffness and; (4) additional CVD risk factors.

1. Common carotid intima-media thickness (IMT)
  External ultrasound of the common carotid artery will be performed with the patient lying in a supine position, neck slightly extended and the head tilted 45° away from the side being scanned whilst connected to 3-lead ECG, to monitor cardiac cycle. Bilateral longitudinal images of the carotid bulb and common carotid artery, up to 10 mm proximal to the bulb, will be obtained using a portable ultrasound (Sonosite, Bothell, USA) with a 10–5 MHz linear array transducer. Both the right and left carotid arteries will each be scanned from three angles, with two loops obtained from each scan angle, and stored in a digital format for batch analysis by an observer blinded to participant characteristics, randomization and study visit. Measurement of carotid IMT will be performed on the far wall of the common carotid artery at end diastole, within 10 mm proximal to the carotid bulb, from at least two consecutive cardiac cycles.
2. Endothelial function—brachial artery flow-mediated dilatation (FMD)
  High-resolution ultrasound will be used to assess brachial artery diameter at rest, and after a hyperaemic stimulus in response to a 5 min forearm occlusion. A 30 s loop will be acquired at rest, and three 30 s loops acquired post-hyperaemia from 30–60 s, 60–90 s, and 90–120 s. Response to sublingual nitroglycerin, a smooth muscle dilator, will not be determined to minimize participant burden.
3. Arterial stiffness—carotid-femoral pulse wave velocity
  Pulse wave velocity along the aortic transit will be assessed using a SphygmoCor XCEL device (Atcor medical). This validated device assesses carotid and femoral pulses concurrently by handheld tonometer and thigh cuff, respectively. Distance from the suprasternal notch to the two sites will be measured immediately after data capture. Two measures of pulse wave velocity will be made, with a 1 minute interval.
4. History and incidence of cardiovascular and cerebrovascular events will be defined as coronary heart disease (myocardial infarction, angina pectoris, coronary revascularisation procedures), ischaemic cerebrovascular disease, peripheral vascular disease and transient ischaemic attack.

Dental assessment: This will include tooth presence, caries experience, periodontal destruction, gingivitis and calculus, plaque, oral mucosal lesions and trauma.

##### Self-report

Self-report information pertaining to socio-demographic, oral health and general health factors will be gathered at baseline.

Socio-demographic factors: These will include age, sex, education level, employment, English-as-first-language, house ownership, number of children and number of people who stayed in house the previous night.

Oral health factors: These will include self-reported oral health, oral health behaviours and oral health-related quality of life.

General health factors: These will include behaviours such as smoking and alcohol consumption, current medication, current diagnoses (for diabetes, cardiovascular disease, hypertension etc.), status of current diagnoses (controlled, uncontrolled etc.) and stress (measured by an adapted PHQ9 scale; a measure of distress that has been validated for Aboriginal adults; [48]).

### Reimbursement for time

As per our standard procedures for Aboriginal participants involved in periodontal interventions, all participants will receive a $AUS20 gift voucher at baseline, 3-, 6-, 12- and 24-month follow-up as reimbursement for time. Each participant will additionally receive free periodontal care and a sample bag of oral health-related items.

### Data analysis

Data analysis will use intention-to-treat and per-protocol approaches, with the effect on primary outcomes based on intention-to-treat analyses. Analyses will follow a general course of descriptive statistics such as distributions and measures of association. Simple cross-tabulations and more elaborate repeated-measures analyses will follow. In brief, the plan for the analysis of each aim is:

- *Aim 1: Extent and severity of periodontal disease compared with national-level counterparts:* Extent of periodontal disease will be defined by the number of periodontal sites in the mouth with a given pocket depth (PD) divided by the number of teeth in the mouth x 100. The National Survey of Adult Oral Health dataset will enable comparison with both general and Aboriginal population-level estimates [49]. General analysis will comprise Chi-square and Student’s t-test within the study sample and non-overlapping 95 % confidence intervals when comparing with population estimates.
- *Aim 2: Changes in cIMT, measures of renal function and CKD/CVD-related death:* The primary analysis will compare changes in cIMT and 95 % confidence intervals. Secondary analyses will examine each outcome variable using separate mixed models, to allow inclusion of all participants without having to impute data. We will use generalised linear mixed models for non-continuous outcomes. Models will include treatment group as a fixed effect. Analyses will be conducted to identify subgroups that modify the response to the intervention (eg. active/passive smoking, socio-economic status). Model assumptions will be checked and appropriate adjustments made. We will conduct sensitivity analyses to assess uncertainty in key parameters. Death and cardiovascular events, both binary outcomes, will be analysed using the chi-square test with frequencies and percentages per treatment arm and odds-ratios or other measures of treatment effect reported along with summary measures. The Greenhouse-Geisser correction for the F-test will be used to adjust the degrees of freedom for deviation from sphericity. Logarithmic transformation of the data will be performed where appropriate. A post hoc correlation analysis by the Spearman rank-correlation method will be performed to evaluate the relationship between the changes in renal function and cIMT from baseline to 6, 12 and 24 months following periodontal therapy. The same will also be done for measures of periodontal health and cIMT. Effect sizes will be calculated by dividing the mean of change scores by the pooled estimate of the standard deviation. Effect measures will also be presented through calculation of number-needed-to-treat and its associated 95 % confidence interval. The statistically significant level will be at α < 0.05.

## Discussion

The proposed study will be the first RCT evaluating the effect of periodontal therapy on progression of CKD and CVD events among Aboriginal patients with CKD. The proposed study will investigate a novel use for a widely available public health service with which every health professional is familiar. This innovative study will provide critical information regarding the progression of renal disease and rates of cardiovascular events among those with CKD, with endpoints used previously by many highly cited RCTs that are international standards used in everyday clinical practice. Demonstration of a significant attenuation of CKD progression and CVD events has the potential to inform clinicians of an important, new and widely available strategy for reducing CKD progression and CVD events for Australia’s most disadvantaged population.

As such, the Perio-CKD study will have significance for policy and planning by providing evidence of the relationship between periodontal therapy and cardiovascular disease among Indigenous Australian adults with chronic kidney disease, and the effectiveness of an intervention aimed at improving periodontal, renal and cardiovascular health in an Indigenous population. Efforts to understand and improve progression of renal disease among Indigenous Australians may serve as an important means of reducing the gap between Indigenous and non-Indigenous health.

The findings may help raise the profile of the role of periodontal disease in cardiovascular and renal health; thus increasing the knowledge base of those working intimately with patients with chronic kidney disease. Ultimately it is hoped that the findings might encourage greater dialogue between oral health and medical professionals so that periodontal treatment might become a routine part of care in the treatment of chronic kidney disease among Indigenous as well as non-Indigenous populations.
